# Supplementary material for: Polyketide synthase gene clusters in the ericoid mycorrhizal fungus Oidiodendron maius: genome-wide prediction, expression profiling, and functional characterization of knockout mutants
Source: Front Plant Sci. 2026 Sep 16;17:1924430. doi: 10.3389/fpls.2026.1924430 (PMC13623903; doi:10.3389/fpls.2026.1924430)
Supplement: Supplementary file 1 [file SupplementaryFile1.zip › Supplementary Materials/Supplementary Folder 1.zip!/Oidma1_AssemblyScaffolds_Repeatmasked/knownclusterblast/cluster7/jgi.p_Oidma1_153340_mibig_hits.html]

| MIBiG Protein | Description | MIBiG Cluster | MiBiG Product | % ID | % Coverage | BLAST Score | E-value |
| --- | --- | --- | --- | --- | --- | --- | --- |
| AJI44188.1 | hypothetical\_protein | BGC0001193 | Thiolutin\_biosynthetic\_gene\_cluster | 43 | 96.1832061069 | 190 | 3.4e-48 |
| AAQ08912.1 | putative\_3-ketoacyl-ACP\_reductase | BGC0000224 | Fredericamycin\_biosynthetic\_gene\_cluster | 36 | 95.4198473282 | 143 | 3.7e-34 |
| ACM68692.1 | putative\_oxidoreductase | BGC0000298 | Aeruginosin\_biosynthetic\_gene\_cluster | 37 | 98.4732824427 | 138 | 1.2e-32 |
| ABS75236.1 | DhbA | BGC0001185 | Bacillibactin\_biosynthetic\_gene\_cluster | 32 | 97.7099236641 | 131 | 1.4e-30 |
| ABB52548.1 | 3-oxoacyl-(acyl-carrier-protein)-reductase | BGC0000047 | Dihydrochalcomycin\_biosynthetic\_gene\_cluster | 35 | 95.0381679389 | 129 | 7.1e-30 |
| BAJ52708.1 | putative\_3-ketoacyl-ACP\_reductase | BGC0000222 | FD-594\_biosynthetic\_gene\_cluster | 38 | 95.4198473282 | 129 | 7.1e-30 |
| ANC94959.1 | AlmK | BGC0001396 | Aldgamycin\_biosynthetic\_gene\_cluster | 36 | 95.0381679389 | 128 | 9.3e-30 |
| ADG86311.1 | 3-ketoacyl\_ACP-reductase | BGC0000190 | A-74528\_biosynthetic\_gene\_cluster | 35 | 95.0381679389 | 128 | 9.3e-30 |
| ACU59350.1 | short-chain\_dehydrogenase/reductase\_SDR | BGC0000839 | Flexirubin\_biosynthetic\_gene\_cluster | 32 | 93.893129771 | 128 | 1.2e-29 |
| AHL46721.1 | ketoreductase | BGC0001179 | Rishirilide\_B\_biosynthetic\_gene\_cluster | 34 | 94.2748091603 | 127 | 2.1e-29 |
| AAQ08926.1 | putative\_3-ketoacyl-ACP\_reductase | BGC0000224 | Fredericamycin\_biosynthetic\_gene\_cluster | 34 | 95.8015267176 | 125 | 7.9e-29 |
| ADG86325.1 | ketoreductase | BGC0000190 | A-74528\_biosynthetic\_gene\_cluster | 32 | 96.9465648855 | 124 | 2.3e-28 |
| AMK51276.1 | Hex19 | BGC0001376 | Hexaricin\_biosynthetic\_gene\_cluster | 34 | 95.8015267176 | 123 | 3.9e-28 |
| ABW11827.1 | short-chain\_dehydrogenase/reductase\_SDR | BGC0001197 | Frankiamicin\_biosynthetic\_gene\_cluster | 33 | 95.0381679389 | 122 | 6.7e-28 |
| CAB15190.2 | 2,3-dihydro-2,3-dihydroxybenzoate\_dehydrogenase | BGC0000309 | Bacillibactin\_biosynthetic\_gene\_cluster | 32 | 97.7099236641 | 120 | 2.5e-27 |
| CAG23976.1 | 3-oxoacyl-(acyl\_carrier\_protein)\_reductase | BGC0000176 | Difficidin\_biosynthetic\_gene\_cluster | 30 | 93.893129771 | 120 | 3.3e-27 |
| CAM58804.1 | BenL\_protein | BGC0000204 | Benastatin\_biosynthetic\_gene\_cluster | 32 | 98.0916030534 | 117 | 2.1e-26 |
| AHX24706.1 | 3-oxoacyl-ACP\_reductase | BGC0000200 | Arixanthomycin\_biosynthetic\_gene\_cluster | 30 | 96.5648854962 | 116 | 6.3e-26 |
| AFO85456.1 | reductase/oxidase | BGC0000391 | Methylpendolmycin/pendolmycin\_biosynthetic\_gene\_cluster | 33 | 95.4198473282 | 115 | 8.2e-26 |
| CAM34340.1 | putative\_3-oxoacyl-ACP\_reductase | BGC0000242 | Lysolipin\_biosynthetic\_gene\_cluster | 31 | 96.5648854962 | 115 | 1.1e-25 |
| AAT12286.1 | LtxD | BGC0000384 | Lyngbyatoxin\_biosynthetic\_gene\_cluster | 32 | 96.5648854962 | 114 | 1.8e-25 |
| ADE22311.1 | putative\_3-oxoacyl-ACP\_reductase | BGC0000279 | Xantholipin\_biosynthetic\_gene\_cluster | 30 | 96.1832061069 | 114 | 2.4e-25 |
| AHX24712.1 | 3-oxoacyl-ACP\_reductase | BGC0000200 | Arixanthomycin\_biosynthetic\_gene\_cluster | 31 | 96.5648854962 | 114 | 2.4e-25 |
| AEI70241.1 | 2,3-dihydro-2,3-dihydroxybenzoate\_dehydrogenase | BGC0000401 | Paenibactin\_biosynthetic\_gene\_cluster | 32 | 97.7099236641 | 114 | 2.4e-25 |
| WP\_029025960.1 | short-chain\_dehydrogenase | BGC0000198 | Arenimycin\_B\_biosynthetic\_gene\_cluster | 32 | 94.2748091603 | 113 | 3.1e-25 |
| ADB23395.1 | dehydrogenase | BGC0001062 | TLN-05220\_biosynthetic\_gene\_cluster | 31 | 95.8015267176 | 112 | 6.9e-25 |
| AGH68923.1 | oxidoreductase | BGC0001083 | Merochlorin\_biosynthetic\_gene\_cluster | 34 | 97.3282442748 | 110 | 3.4e-24 |
| ADE22321.1 | 3-oxoacyl-ACP\_reductase | BGC0000279 | Xantholipin\_biosynthetic\_gene\_cluster | 30 | 96.1832061069 | 110 | 3.4e-24 |
| AAG03070.2 | RubF | BGC0000266 | Rubromycin\_biosynthetic\_gene\_cluster | 31 | 96.1832061069 | 109 | 4.5e-24 |
| CAD62198.1 | Ata4\_protein | BGC0000873 | A201A\_biosynthetic\_gene\_cluster | 32 | 93.5114503817 | 108 | 1.3e-23 |
| AET51861.1 | 3-ketoacyl\_ACP\_reductase | BGC0001138 | A201A\_biosynthetic\_gene\_cluster | 30 | 93.1297709924 | 108 | 1.3e-23 |
| CAM34370.1 | putative\_3-oxoacyl-ACP\_reductase | BGC0000242 | Lysolipin\_biosynthetic\_gene\_cluster | 29 | 95.0381679389 | 107 | 2.2e-23 |
| ABM21753.1 | PdmG | BGC0000256 | Pradimicin\_biosynthetic\_gene\_cluster | 29 | 96.5648854962 | 107 | 2.9e-23 |
| CBZ42141.1 | Acyl-Co\_Adehydrogenase | BGC0001117 | Himastatin\_biosynthetic\_gene\_cluster | 30 | 108.396946565 | 106 | 3.8e-23 |
| ACX83621.1 | keto\_reductase | BGC0000221 | Erdacin\_biosynthetic\_gene\_cluster | 31 | 96.1832061069 | 106 | 5e-23 |
| EWM63056.1 | 2,3-dihydro-2,3-dihydroxybenzoate\_dehydrogenase | BGC0000679 | Diazepinomicin\_biosynthetic\_gene\_cluster | 31 | 98.8549618321 | 106 | 6.5e-23 |
| AAM33685.1 | putative\_bifunctional\_cyclase/3-oxoacyl-ACP\_reductase\_GrhT | BGC0000230 | Griseorhodin\_biosynthetic\_gene\_cluster | 30 | 97.3282442748 | 105 | 1.1e-22 |
| ABP53501.1 | short-chain\_dehydrogenase/reductase\_SDR | BGC0001041 | Salinosporamide\_biosynthetic\_gene\_cluster | 29 | 97.3282442748 | 104 | 2.5e-22 |
| ABP73642.1 | SalM | BGC0000145 | Salinosporamide\_A\_biosynthetic\_gene\_cluster | 29 | 97.3282442748 | 104 | 2.5e-22 |
| AAG30255.1 | NADPH-dependent\_acetoacetyl\_CoA\_reductase | BGC0000867 | Polyhydroxyalkanoic\_acids\_biosynthetic\_gene\_cluster | 34 | 70.2290076336 | 103 | 3.2e-22 |
| ABC42552.1 | putative\_3-ketoacyl\_ACP\_reductase | BGC0000698 | Hygromycin\_A\_biosynthetic\_gene\_cluster | 29 | 93.893129771 | 102 | 5.5e-22 |
| AAF23366.1 | PhaB | BGC0000866 | Polyhydroxyalkanoate\_biosynthetic\_gene\_cluster | 32 | 95.8015267176 | 102 | 5.5e-22 |
| AAD37452.1 | putative\_ketoacyl\_reductase | BGC0000252 | Nonactin\_biosynthetic\_gene\_cluster | 30 | 95.4198473282 | 98 | 1.3e-20 |
| ABD65959.1 | acyl-CoA\_dehydrogenase/reductase | BGC0000341 | Enduracidin\_biosynthetic\_gene\_cluster | 29 | 106.488549618 | 98 | 1.3e-20 |
| ADI58630.1 | AsuB3 | BGC0000187 | Asukamycin\_biosynthetic\_gene\_cluster | 32 | 97.7099236641 | 97 | 3e-20 |
| CAG14976.1 | putative\_3-keto-acyl\_carrier\_protein\_reductase | BGC0000253 | Oviedomycin\_biosynthetic\_gene\_cluster | 31 | 99.6183206107 | 90 | 2.8e-18 |
| ABY83162.1 | Azi24 | BGC0000960 | Azinomycin\_B\_biosynthetic\_gene\_cluster | 29 | 104.198473282 | 89 | 4.8e-18 |
| CUW01207.1 | putative\_3-oxoacyl-ACP\_reductase | BGC0001359\_c2 | PM100117\_/\_PM100118\_biosynthetic\_gene\_cluster | 30 | 102.290076336 | 89 | 6.3e-18 |
| ADD83005.1 | PtnO7 | BGC0001156 | Platencin\_biosynthetic\_gene\_cluster | 31 | 97.3282442748 | 88 | 1.1e-17 |
| ABI75108.1 | short-chain\_alcohol\_dehydrogenase | BGC0000188 | Saxitoxin\_biosynthetic\_gene\_cluster | 35 | 77.0992366412 | 87 | 2.4e-17 |
| BAH33405.1 | 2,3-dihydro-2,3-dihydroxybenzoate\_dehydrogenase | BGC0000371 | Heterobactin\_biosynthetic\_gene\_cluster | 28 | 98.8549618321 | 84 | 2.6e-16 |
| ACO31293.1 | PtmO7 | BGC0001140 | Platensimycin\_/\_platencin\_biosynthetic\_gene\_cluster | 31 | 97.7099236641 | 83 | 4.5e-16 |
| CBK62720.1 |  | BGC0001115 | Thiomarinol\_biosynthetic\_gene\_cluster | 25 | 93.1297709924 | 81 | 1.3e-15 |
| AHZ20771.1 | 3-oxoacyl-acyl-carrier-protein\_reductase | BGC0000369 | Hassallidins\_biosynthetic\_gene\_cluster | 31 | 99.6183206107 | 81 | 2.2e-15 |
| AAM12932.1 | MupS | BGC0000182 | Mupirocin\_biosynthetic\_gene\_cluster | 26 | 92.7480916031 | 79 | 8.5e-15 |
| AHL46696.1 | ketoreductase | BGC0001177 | Mensacaricin\_biosynthetic\_gene\_cluster | 31 | 104.580152672 | 77 | 1.9e-14 |
| AAQ84160.1 | ChcA | BGC0000123 | Phoslactomycin\_B\_biosynthetic\_gene\_cluster | 28 | 93.893129771 | 77 | 2.5e-14 |
| EDY47129.1 | conserved\_hypothetical\_protein | BGC0000845 | Clavulanic\_acid\_biosynthetic\_gene\_cluster | 29 | 74.8091603053 | 77 | 3.2e-14 |
| ACH85578.1 | CetF1 | BGC0000283 | Cetoniacytone\_A\_biosynthetic\_gene\_cluster | 26 | 101.145038168 | 76 | 5.5e-14 |
| BAK64641.1 | dihydroxy\_ketone\_synthase | BGC0000135 | Reveromycin\_biosynthetic\_gene\_cluster | 26 | 105.72519084 | 72 | 1e-12 |
| BAR73014.1 | putative\_3-ketoacyl-ACP\_reductase | BGC0001194 | Hitachimycin\_biosynthetic\_gene\_cluster | 29 | 105.34351145 | 67 | 2.6e-11 |
| ABC42563.1 | putative\_short\_chain\_dehydrogenase | BGC0000698 | Hygromycin\_A\_biosynthetic\_gene\_cluster | 26 | 104.961832061 | 64 | 2.8e-10 |
| AID65226.1 | short\_chain\_dehydrogenase | BGC0000335 | Cystomanamides\_biosynthetic\_gene\_cluster | 30 | 79.3893129771 | 61 | 1.8e-09 |
| BAO66523.1 | carveol\_dehydrogenase | BGC0000042 | Cremimycin\_biosynthetic\_gene\_cluster | 24 | 106.870229008 | 59 | 5.3e-09 |
